# Treatment of *Dientamoeba fragilis*: A retrospective Finnish analysis of faecal clearance and clinical cure comparing four antiprotozoal drugs

**DOI:** 10.1016/j.nmni.2023.101179

**Published:** 2023-09-23

**Authors:** Jukka-Pekka Pietilä, Tuuve A Häkkinen, Laura Pakarinen, Jukka Ollgren, Anu Kantele

**Affiliations:** a Meilahti Vaccine Research Center MeVac, Department of Infectious Diseases, University of Helsinki and Helsinki University Hospital, Helsinki, Finland; b Human Microbiome Research Program, Faculty of Medicine, University of Helsinki, Helsinki, Finland; c Department of Infectious Diseases, Inflammation Center, Helsinki University Hospital, Helsinki, Finland; d Department of Social Services and Health Care, City of Helsinki, Finland; e Department of Health Security, Finnish Institute for Health and Welfare, Helsinki, Finland; f Finnish Multidisciplinary Center of Excellence in Antimicrobial Resistance Research, FIMAR, University of Helsinki, Helsinki, Finland

**Keywords:** *Dientamoeba fragilis*, Parasite, Stool pathogen, Paromomycin, Metronidazole, Secnidazole, Doxycycline, Diarrhoea, Chronic diarrhoea, Stomachache

## Abstract

**Background:**

*Dientamoeba fragilis* (*DF*), the most common intestinal protozoal pathogen in affluent countries, causes asymptomatic or symptomatic infections with severity ranging from mild to disabling. Currently, many studies of treatment options only have small sample sizes and report results that are partly contradictory.

**Methods:**

Investigating data retrieved from Helsinki University Hospital and Helsinki City patient records, we searched for the most effective antiprotozoal in treating *DF* infections. To study microbiological clearance of *DF*, we collected laboratory results of control samples from patients given one of four commonly used antiprotozoals: doxycycline, metronidazole, paromomycin, or secnidazole. For patients symptomatic prior to antiprotozoal treatment, we also retrieved data on clinical outcomes. Furthermore, we explored factors associated with faecal clearance and clinical cure.

**Results:**

A total of 369 patients (median age 38) and 492 treatment episodes were included. Paromomycin (n ​= ​297) proved effective (clearance rate 83%), showing strong association with faecal clearance (aOR 18.08 [7.24–45.16], p ​< ​0.001). For metronidazole the rate was 42% (n ​= ​84), for secnidazole 37% (n ​= ​79), and doxycycline 22% (n ​= ​32). In pairwise comparisons, paromomycin outdid the three other regimens (p ​< ​0.001, *χ*^*2*^ test). Faecal clearance was associated with clinical cure (aOR 5.85 [3.02–11.32], p ​< ​0.001).

**Conclusions:**

Faecal clearance, strongly associated with clinical cure, is most effectively achieved with a course of paromomycin, followed by metronidazole, secnidazole and doxycycline. Our findings will be useful in devising treatment guidelines for adults with symptomatic *D. fragilis* infection.

## Highlights

• In *Dientamoeba fragilis* infection, faecal clearance is associated with clinical cure.
• Faecal clearance is best achieved with paromomycin.
• Paromomycin is superior over metronidazole, doxycycline and secnidazole.
• Doxycycline is not a drug of choice in treatment of *Dientamoeba fragilis* infection.
• Paromomycin is preferable as first-line medication in eradicating *D. fragilis*.

## 1 Introduction

*Dientamoeba fragilis,* a common intestinal protozoan has a worldwide distribution [1,2], with prevalences ranging from 11% [3] to 71% [4] between studies, depending on study population, design and setting [5]. While some investigators consider *DF* commensal [6,7] mainly arguing the high prevalence of asymptomatic infections, others describe gastrointestinal symptoms which vary from mild to disabling in severity [1,2], and many report disappearance of symptoms after successful clearance, particularly among adults [2,[8], [9], [10], [11], [12]]. Even to date, *D. fragilis* appears to be underdiagnosed and its treatment often delayed [13].

Research into the treatment of *DF* infection remains scarce and sample sizes small. A variety of antimicrobials are known to have efficacy against *DF*, including 5-nitroimidazole derivatives, aminoglycosides, iodoquinol, and tetracyclines [14], with clearance rates ranging from 55% to 100% [15,16]. The few comparisons conducted between the drugs [15,[17], [18], [19]] mostly look at children, sometimes yielding rather contradictory results.

In 2019, we showed *DF* to be the most common pathogenic intestinal parasite in the Helsinki Metropolitan Area in Finland [2]. We found that clinical improvement was associated with faecal clearance, consistent with some other studies [3,8,10,11]. Spurred by our results, we now studied a considerably larger patient cohort, covering both microbiological clearances and clinical successes separately for the four antiprotozoal most commonly used in Finland: doxycycline, metronidazole, paromomycin, and secnidazole.

## 2 Materials and methods

### 2.1 Study outline

We compared doxycycline, metronidazole, paromomycin, and secnidazole in treatment of *DF* infection within the Helsinki Metropolitan area between January 2007 and December 2016. First, we identified all patients with *DF* in the laboratory database and selected those given these medications. Next, to analyse faecal clearance and clinical cure, we explored the pre- and post-treatment data. Microbiological clearances were compared pairwise between the drugs, with separate analyses for subgroups of adults and patients aged <18 years.

The study protocol was approved by the Department of Medicine and the HUSLAB laboratory at the Helsinki University Hospital (HUH) and the Department of Social Services and Health Care in the City of Helsinki. According to the Finnish Medical Research Act, a review by the Ethical Committee was not required, as this study did not involve interventions (Ministry of Social Affairs and Health, Finland: Medical Research Act. 2015 Jan 14; 1–5).

### 2.2 Data collection and handling of specimens

From the HUSLAB database we retrieved data on patients with DF in microscopy or PCR. Faecal sample for microscopy was fixed in Ecofix® (Meridian Bioscience, Inc., Cincinnati, United States) immediately after defecation and subjected to modified trichrome staining (later *trichrome sample*) [20]. Faecal samples in eNAT tubes were analysed by RT-PCR (Amplidiag® Stool Parasites test, Mobidiag Ltd, Finland) [21]. During the study period, faecal PCR was only sporadically employed in routine diagnostics.

### 2.3 Selection of patient population

We included all patients with medical records available between January 2007 and December 2016, except those with 1) concurrent faecal pathogens other than *DF*; 2) an active gastrointestinal disease diagnosed; 3) other *DF* medication than any of the four drugs examined, or simultaneous use of multiple antimicrobials; 4) post-treatment control faecal samples tested by methods other than trichrome or PCR; 5) post-treatment control samples not provided 14–90 days after last dose; or 6) only one trichrome control sample delivered (with PCR, a single control sample sufficed).

### 2.4 Background information and clinical data

From the electronic medical records of HUH and the City of Helsinki, we collected information on age, sex, ethnicity, underlying diseases, and recent travel history (within one year before diagnosis) and results of other stool microbe analyses, positive findings leading to exclusion. All pre- and post-treatment symptom data were retrieved; symptoms not mentioned were interpreted as absent.

### 2.5 Faecal clearance and clinical cure

The dosages of all four regimens were recorded. For patients with several medications, each course was included as one episode.

Faecal clearance and clinical cure were evaluated separately and analysed for associations. Clearance was considered successful if at least two post-treatment trichrome samples (or one PCR sample) had been recorded as negative and none of the other possible control samples proved positive (trichrome or PCR). Only episodes with control samples provided 14–90 days after the last treatment day were included.

Clinical cure was defined as complete symptom resolution. In analyses of the rates of clinical cure, we only included patients initially symptomatic.

### 2.6 Statistics

The distribution of continuous variables was described by medians and interquartile ranges. For categorical variables distributions and 95% confidence intervals were determined. The *χ*^*2*^ test was used for comparisons; the *Fisher's exact test* was applied for small sample sizes. Significance levels were Bonferroni-adjusted in multiple comparison situations. Factors associated with faecal clearance and clinical cure were determined by a multivariable binary logistic regression model. Statistical analyses were conducted using SPSS software version 25 (IBM, New York, United States).

## 3 Results

### 3.1 Subject group

**Fig. 1:**
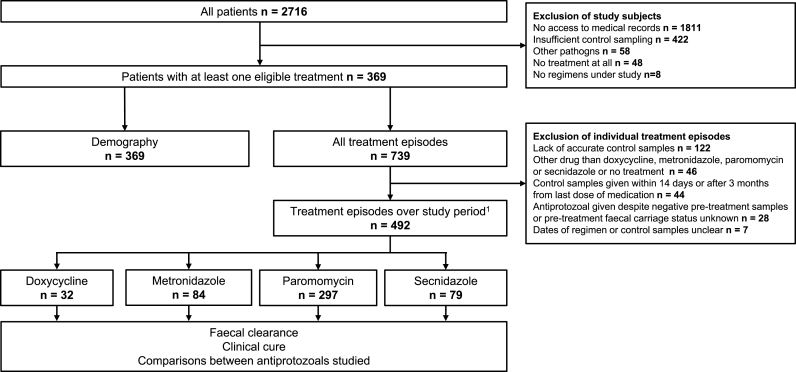
Study design.

We identified 2716 patients with a positive *DF* finding in trichrome or PCR samples recorded in the HUSLAB database over the study period. After exclusions (1811 for missing medical records; 422 for insufficient control sampling; 58 for other pathogens; 48 for no treatment; 8 for no regimens studied here), 369 patients with *DF* comprised the study population (Fig. 1).

### 3.2 Demographics and microbiological samples

Of the 369 patients, 63% were female and 88% of Finnish ethnic origin. The median age was 38.0 years (with no differences between genders), and 26% were aged <18 years; 54% had travelled abroad the preceding year (Table 1). Of all patients, 88% (323/369) were symptomatic (Supplementary Table 1). For other stool pathogens and apathogenic parasites, see Supplementary Tables 2 and 3. Supplementary Table 4 presents the demographic data in more detail.

**Table 1:** Demographics of 369 patients diagnosed with *Dientamoeba fragilis* infection between 2007 and 2016 in Helsinki and Uusimaa Hospital District, Finland.

Variable	Number (%)
Gender	
Female	233 (63)
Male	136 (37)
Age, median (IQR)	38 (15.1–51.0)
Males, median (IQR)	40.0 (11.0–48.9)
Females, median (IQR)	38.0 (21.0–52.6)
≤ 17 years	95 (26)
18–35 years	72 (20)
36–55 years	122 (33)
≥ 56 years	80 (22)
Immigrant	41 (11)
Pre-existing conditions	161 (44)
Gastrointestinal disorders	57 (15)
Lactose intolerance or IBS	26 (7)
Coeliac disease or IBD	10 (3)
GERD	7 (2)
Other	14 (4)
Cardiovascular diseases	46 (12)
Autoimmune, immunodeficiency and haematological disorders including HIV infection and cancer	39 (11)
Asthma, atopy and/or allergy	37 (10)
Other^a^	30 (8)
Endocrinological disorders	28 (8)
Foreign travel within 12 months	200 (54)
Tropics or subtropics	88 (24)
Other parasites^b^	208 (56)
Number of individual treatments	492
Doxycycline	32
Age (years), median (IQR)	44.5 (35.0–64.3)
Metronidazole	84
Age (years), median (IQR)	40 (16.0–51.5)
Paromomycin	297
Age (years), median (IQR)	42 (30.0–56.0)
Secnidazole	79
Age (years), median (IQR)	8.7 (5.0–12.5)

a E.g. musculoskeletal 10 (2), neurological 6 (2) and psychiatric 5 (2) disorders, and other illnesses classified into disciplines not mentioned in table.

b Other intestinal parasites comprise *Blastocystis*, *Entamoeba coli* and *hartmannii*, *Endolimax nana*, *Chilomastix mesnili*, and *Iodamoeba butschlii*.

### 3.3 Treatment

#### 3.3.1 Treatment episodes and control samples

The 369 *DF* patients had been given antiprotozoals for a total of 739 episodes, 492 of which were included in the treatment analyses (for exclusions, see Fig. 1). Paromomycin was used in 60%, metronidazole 17%, secnidazole 16%, and doxycycline in 6% of the episodes. The dosages proved consistent, and for 86% (422/492) of the treatment episodes all data were available (Supplementary Table 4).

The median time was 30 days (IQR 25–43) for first control sample after last treatment day and 49 days (IQR 30–77) for the second (Supplementary Table 4). In subgroup analysis, the first control samples were provided slightly earlier by those given paromomycin than those receiving the other regimens. However, no difference was seen in the median time of the second control samples (Supplementary Table 4).

Of the treatment episodes, 73% (361/492) were reported for adult patients and 27% (131/492) for those aged <18 years; 53% (69/131) of the latter included secnidazole. The regimens used varied by age group, with secnidazole commonly administered to those <18 years, but rarely to adults. Doxycycline, metronidazole, and paromomycin were used almost entirely for adults (Supplementary Table 4).

#### 3.3.2 Faecal clearance

**Fig. 2:**
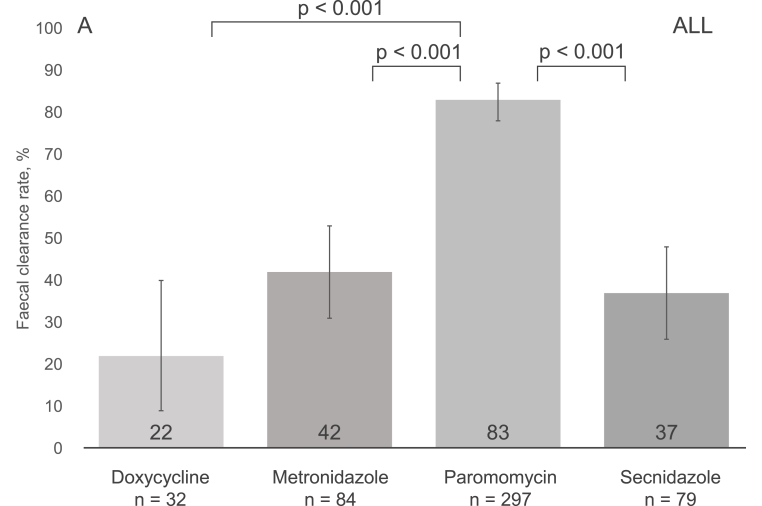

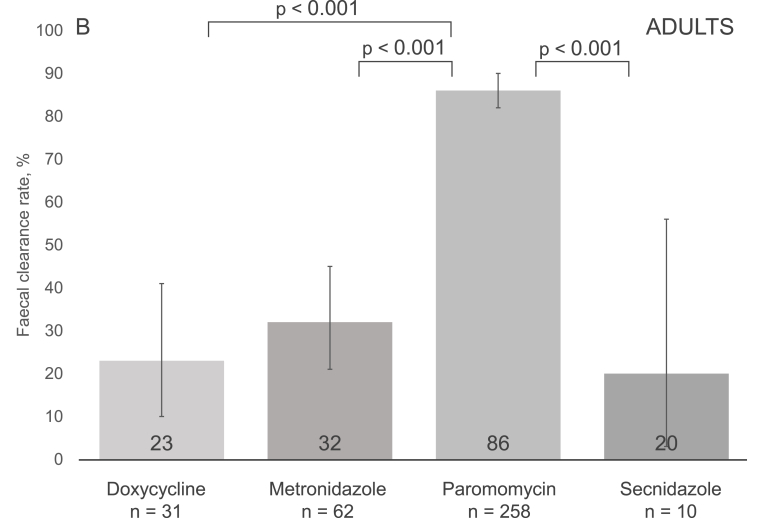

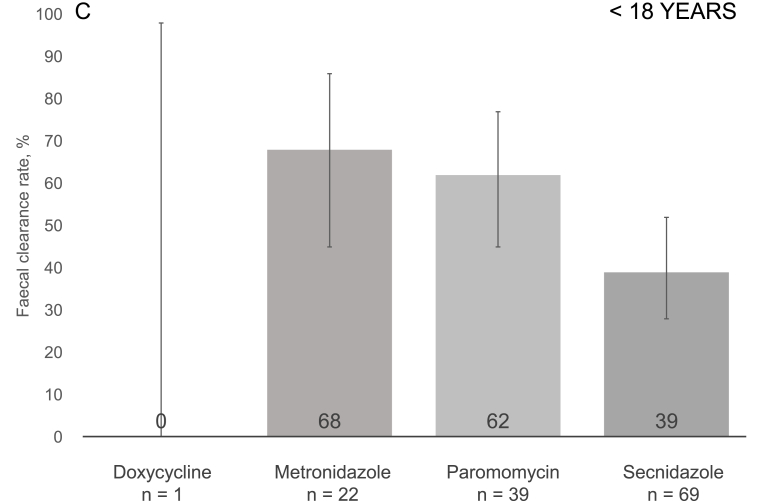
Post-treatment faecal eradication rates for all participants (Panel A), adults (Panel B), and those aged <18 years (Panel C). P-values for pairwise comparison of faecal clearances of each regimen were obtained using the χ^2^ test. Significance level was Bonferroni adjusted with the number of comparisons (6) to 0.008. Only statistically significant comparisons are illustrated, please see Supplementary Table 6 for other p-values. Panel A. The 95% confidence intervals were as follows: doxycycline 9.3–40.0, metronidazole 26.1–48.3, paromomycin 78.4–87.2, and secnidazole 26.1–48.3. Panel B. The 95% confidence intervals were as follows: doxycycline 9.6–41.1, metronidazole 20.9–45.3, paromomycin 81.6–90.4, and secnidazole 2.5–55.6. Panel C. The 95% confidence intervals were as follows: doxycycline 0.0–97.5, metronidazole 45.1–86.1, paromomycin 44.6–76.6, and secnidazole 27.6–51.6.

The overall faecal clearance rate for all treatment episodes reached 65% (318/492), with the highest rates for paromomycin at 83% (247/297), followed by metronidazole at 42% (35/84), secnidazole at 37% (29/79), and doxycycline at 22% (7/32) (Fig. 2, panel A). In pairwise comparisons, paromomycin showed a significantly higher clearance rate than any of the other drugs; p ​< ​0.001 (*χ2* test, adjusted significance level p ​≤ ​0.008) for each comparison (Fig. 2, panel A). In subgroup analyses, differences were seen between courses prescribed for adults (Fig. 2, panel B), but not patients <18 years old (Fig. 2, panel C). The clearance rates did not vary significantly by order of course (first, second or third) (Supplementary Table 5).

In our binary regression model, paromomycin treatment was associated with faecal clearance (p ​< ​0.001). Furthermore, metronidazole use and male gender appeared to be associated, but without reaching statistical significance (p-values 0.07 and 0.08, respectively). No association was found for the other factors studied (age, sex, ethnic origin, apathogenic parasite carriage, foreign travel, pre-existing conditions) (Table 2).

**Table 2:** Regression analysis of factors associated with faecal clearance and clinical cure after individual antiprotozoal treatment episodes.

Variables	Faecal clearance (n = 476)^a^		Clinical cure (n = 369)^b^	
	OR (95% CI)	p-value	OR (95% CI)	p-value
Treatment		< 0.001		0.17
Metronidazole	2.49 (0.94–6.59)	0.07	0.62 (0.18–2.11)	0.45
Paromomycin	18.08 (7.24–45.16)	< 0.001	0.71 (0.23–2.24)	0.56
Secnidazole	1.95 (0.67–5.65)	0.22	0.26 (0.06–1.04)	0.06
Doxycycline	Ref	N/A	Ref	N/A
Successful faecal clearance^c^	N/A	N/A	5.84 (3.02–11.31)	< 0.001
Gender				
Male	1.53 (0.95–2.45)	0.08	1.46 (0.88–2.41)	0.14
Age	1.00 (0.99–1.01)	0.96	0.97 (0.96–0.99)	< 0.001
Ethnic origin				
Non-Finnish	0.93 (0.43–2.01)	0.86	1.05 (0.45–2.46)	0.91
Having pre-existing conditions	1.13 (0.67–1.73)	0.65	1.37 (0.79–2.40)	0.26
Gastrointestinal illnesses	0.86 (0.42–1.78)	0.69	0.77 (0.37–1.61)	0.50
Carriage of other parasites		0.52		0.84
*Blastocystis*	0.93 (0.58–1.50)	0.76	1.12 (0.67–1.87)	0.67
Other than *Blastocystis*	0.62 (0.27–1.41)	0.25	1.27 (0.50–3.20)	0.62
Foreign travel within 12 months	1.15 (0.72–1.83)	0.56	1.18 (0.70–1.97)	0.54
Symptoms prior to treatment	0.79 (0.43–1.43)	0.43	N/A	N/A

All *treatment x other variable* interactions were non-significant (p > 0.07) in clinical cure analysis. In clearance analysis age × treatment interaction had a p < 0.001 but was left out of our model for clinical insignificance; all *treatment x other variable* interactions were non-significant (p > 0.07).

a Faecal clearance analysis is missing 13 paromomycin and 3 secnidazole episodes, as there were no data to show, whether the patients were symptomatic or not before medication.

b All patients were symptomatic prior to treatment.

c Successful faecal clearance was defined by negative post-treatment samples.

Despite significant demographic differences between the various treatment groups (Supplementary Table 4), the results of the best fitted model did not provide any significant evidence of interactions between treatment groups and other variables (Table 2).

#### 3.3.3 Association between faecal eradication and clinical cure

Regression analysis associated faecal clearance with clinical cure (OR 5.85 [3.02–11.32], p ​< ​0.001) (Table 2). Furthermore, individual analyses of our four antiprotozoals showed a significant association between faecal clearance and clinical cure for metronidazole (OR 22.96 [4.06–129.75], p ​< ​0.001), and paromomycin (OR 2.48, 1.21–5.08, p ​= ​0.001), but not secnidazole (OR 1.45 [0.46–4.60], p ​= ​1.000) mostly prescribed for children (Supplementary Table 5). While not tested by regression analysis (small sample size), *χ*^*2*^ test detected an association also for doxycycline (p ​= ​0.001) (Table 3).

**Table 3:** Clinical outcome after successful faecal clearance of symptomatic *D. fragilis* infection using doxycycline, metronidazole, paromomycin, or secnidazole. Test of significance with χ^2^ test. (Comparison between episodes with faecal clearance and no clearance.)

	Treatment episodes								
Faecal clearance rates	All			Adults			<18 years		
	Cured	Not cured	p-value (comparison between clearance and no clearance)	Cured	Not cured	p-value (comparison between clearance and no clearance)	Cured	Not cured	p-value (comparison between clearance and no clearance)
**All episodes, n**	129	240	<0.001	94	194	<0.001	35	46	0.004
Faecal clearance, n (%)	113 (88)	131 (55)		88 (94)	113 (58)		25 (71)	18 (39)	
**Doxycycline, n**	6	17	0.001^a^	6	16	0.001^a^	N/A	N/A	N/A
Faecal clearance, n (%)	5 (83)	1 (6)		5 (83)	1 (6)		N/A	N/A	
**Metronidazole, n**	21	57	<0.001	9	50	0.018^a^	12	7	0.01^a^
Faecal clearance, n (%)	17 (81)	14 (25)		6 (67)	12 (24)		11 (92)	2 (29)	
**Paromomycin, n**	91	129	0.001	78	122	0.001	13	7	0.035^a^
Faecal clearance, n (%)	87 (96)	102 (79)		76 (97)	100 (82)		11 (85)	2 (29)	
**Secnidazole, n**	11	37	1.000^a^	1	6	0.143^a^	10	31	0.480^a^
Faecal clearance, n (%)	4 (37)	14 (38)		1 (100)	0 (0)		3 (30)	14 (45)	

Only symptomatic patients' treatment episodes were included. Asymptomatic patients were omitted from analyses.

The control samples totalled 1110, of which 98.9% (1098/1110) were trichrome stained and 1.1% (12/1110) analysed by faecal PCR.

a P-values by *2-sided Fisher's Exact* test.

In subgroup analyses by age group, a significant association was seen for adults (OR 11.55 [4.08–32.69], p ​< ​0.001). Among those aged <18 years, clearance rate and clinical cure appeared to be associated, but the difference did not reach statistical significance (OR 2.94 [0.87–9.95], p ​= ​0.082). Unfortunately, due to small sample sizes, regression analysis by individual antiprotozoals was not feasible for separate scrutiny of adults and those <18 years of age.

## 4 Discussion

To explore the various antiprotozoals used in treating *DF* infections, we scrutinized faecal clearance rates and association with clinical cure. We report two major results: 1) in clearing out the pathogen, paromomycin outdoes three other antiprotozoals, doxycycline, metronidazole, and secnidazole; 2) enabled by a large cohort, our previous finding of an association between faecal clearance and clinical cure is further reinforced, especially for adults.

### 4.1 Overall faecal clearance

To our knowledge, our study scrutinises in a single design the largest data to date on *Dientamoeba fragilis* episodes treated with paromomycin (n ​= ​297), secnidazole (n ​= ​79), and doxycycline (n ​= ​32). The clearance rate for paromomycin exceeds those for doxycycline, metronidazole, and secnidazole (p ​< ​0.001 against each). This confirms previous reports of paromomycin's superiority over metronidazole [16,17,19].

Paromomycin's clearance rate of 83% accords with earlier results varying between 78% and 100% [3,9,10,16,17,19,22]. All other antiprotozoals yielded somewhat weaker results than previously reported. In our data, doxycycline only reached a rate of 22% (n ​= ​32). In the literature, data are available only on individual patients, reporting clearance for three out of four [23] and a single individual [24]. Likewise, our 37% rate for secnidazole remains far below the 97% found earlier [8] in spite of our greater number of treatment episodes (79 versus 34). Our 42% faecal clearance rate for metronidazole fell short of those in previous studies ranging between 53% and 85% [3,6,9,11,12,15,[17], [18], [19]].

Our lower clearance rates could be ascribed to bigger sample sizes and, partly, timing of controls: we collected specimens 34 and 45 days (medians) after the last dose, while, as exemplified by one study of secnidazole, control sampling has in many research designs been timed for such days as 7 and 14 [8]. Indeed, at least with metronidazole among day care-aged children, the clearance rate has been reported to decrease from 62.5% at two weeks to 24.9% at eight weeks after treatment [6] pointing to lower rates in later control sampling – and speaking against spontaneous eradication of *DF*. The decrease in clearance may reflect substantial reinfection rates rather than recrudescence: day-care aged children are at high risk of *DF* carriage, and the pathogen appears to be easily transmitted within families [25].

### 4.2 Association between faecal clearance and clinical cure

Faecal clearance was associated with clinical cure, our finding according with numerous previous investigations [1,2,[8], [9], [10], [11], [12]]. Our results contradict a double-blinded randomised paediatric study reporting no association between clearance and clinical cure [6]. However, methodological criticism points to those results not having been conclusive [26]. Nor was any association reported between *DF* carriage and symptoms in some later studies among children [[27], [28], [29]], the data consistent with high asymptomatic carriage rates for this age group [7,30]. To further explore potential differences between adults and children, we separately analysed the data within the two age groups. This scrutiny revealed an association between faecal clearance and clinical cure that reached statistical significance among adults, but not patients aged <18 years.

It is noteworthy that for some patients with successful faecal clearance, symptoms only wane gradually – a pattern also seen in giardiasis. Since we classified eased symptoms as clinical failure, the clinical cure rates probably remain underestimates.

### 4.3 Background for guidelines

Our data serve as background information for devising international/local treatment guidelines. While no international guidelines care currently available, the Center for Disease Control and Prevention has suggested paromomycin, iodoquinol, and metronidazole as the primary medication [31]. In the Netherlands, one clinical guideline favours clioquinol as first-line drug [5] despite its associated central nervous system adverse events. Since the current data have already been implemented into the Finnish guidelines, paromomycin is recommended as first-line treatment, whenever available. This choice is supported by studies showing it to be well tolerated [32]. If paromomycin is not available, metronidazole or secnidazole are, despite their lower efficacy, the drugs of choice. Treating patients with no symptoms is not generally warranted. As obvious, despite eradication of *DF*, not all patients will be cured: abdominal complaints may – partly or fully – have some other aetiology. On the other hand, early treatment of symptomatic *D. fragilis* patients may help to avoid more extensive diagnostic examinations.

### 4.4 Limitations

Because of our retrospective design, data on all gastrointestinal pathogens were not available for all participants. However, since prolonged diarrhoea is particularly characteristic of parasitic pathogens, the most common of them (*Giardia* and *Cryptosporidium* spp.) were by default covered for all participants using either trichrome samples or faecal PCR test. On the other hand, in prolonged diarrhoea viral aetiology is presumed rare [33]; bacterial aetiology had been excluded for 43%. We could not include a ‘no treatment’ control group, because such patients usually lacked control samples. As microbiological results may impact symptom evaluation, we recommend the clinical approach adopted by our department: results are given only after the clinical outcome has been recorded.

We acknowledge that fixation-trichrome staining is less sensitive than PCR [34,35]. Comparisons between the various regimens and individuals were nevertheless valid, for the same method was used in pre- and post-treatment analyses. Furthermore, as the clearance rates between episodes with two or three control samples did not differ significantly (data not shown), we chose not to require the recommended three control samples [36] for inclusion.

### 4.5 Conclusions

Our study shows a significant association between faecal clearance and clinical cure, a finding strongly encouraging medical therapy for symptomatic patients. Demonstrating its superior efficacy over metronidazole, secnidazole and doxycycline, our results support the use of paromomycin as the first-line antiprotozoal. Doxycycline should not be considered a drug of choice at all.

## Authors’ contributions

Pietilä Jukka-Pekka: conceptualisation, methodology, formal analysis, investigation, data curation, writing – original draft, visualisation, project administration, funding acquisition; Häkkinen Tuuve A: formal analysis, writing – review, editing; Pakarinen Laura: resources, writing – review, editing; Ollgren Jukka: formal analysis, writing – review, editing; Kantele Anu: conceptualisation, methodology, resources, writing – original draft, visualisation, supervision, funding acquisition.

## Funding

This work was supported by a Finnish government subsidy for health science research [grant numbers: TYH 2012141, TYH 2013218 and TYH 2014216], the Sigrid Jusélius Foundation [grant number: 1726], the 10.13039/501100003125Finnish Cultural Foundation [grant number: 00200853] and the Doctoral Programme in Clinical Research, Doctoral School in Health Sciences, 10.13039/100007797University of Helsinki.

## Declaration of competing interest

The authors declare that they have no known competing financial interests or personal relationships that could have appeared to influence the work reported in this paper.
