# Supplementary material for: Treatment of Dientamoeba fragilis: A retrospective Finnish analysis of faecal clearance and clinical cure comparing four antiprotozoal drugs
Source: New Microbes New Infect. 2023 Sep 23;54:101179. doi: 10.1016/j.nmni.2023.101179 (PMC10542007; doi:10.1016/j.nmni.2023.101179)
Supplement: Multimedia component 1 [file mmc1.docx]

**SUPPLEMENTARY MATERIAL**

**Supplementary Table 1.** Symptoms among 369 *Dientamoeba fragilis*-infected patients.*

| **Symptom** | **Number reported, n (%)** |
| --- | --- |
| Any | 323 (88) |
| Loose stools or diarrhoea | 231 (63) |
| Abdominal pain | 205 (56) |
| Flatulence | 132 (36) |
| Abdominal bloating | 76 (21) |
| Weight loss | 68 (18) |
| Nausea | 63 (17) |
| Fatigue | 43 (12) |
| Vomiting | 41 (11) |
| Faecal urgency | 36 (10) |
| Fever (temp ≥ 37,5°) | 32 (9) |
| Constipation | 30 (8) |

*88 % of patients were explored for *DF* on account of typical symptoms. 12 % (46/369) were asymptomatic: 26 had been screened because of a symptomatic family member’s suspected reinfection, 11/46 because of peripheral eosinophilia, 1 because of anaemia. For 7 patients the reason for examination had not been recorded.

**Supplementary Table 2.** Microbiological findings from faecal sample analyses. NB Findings for pathogens resulted in exclusion, while those of apathogens did not.

| **Faecal sample or analysis** | **Number of tests conducted** | **Pathogen findings (% of samples)^1^** | **Number of sampled patients in final subject group (%)^2^** |
| --- | --- | --- | --- |
| Trichrome sample^3^ | 881 | *Giardia* *lamblia* 12 (1)  *Entamoeba histolytica* 2 (0) | 368 (99)^4^ |
| Formalin sample for enteric parasites, ova and worms | 740 | *G. lamblia* 11 (1)  *Hymenolepsis nana* 3 (0)  *E. histolytica* 2 (0)  *Cryptosporidium* spp. 1 (0)  *Trichuris trichiura* 1 (0)  *Ascaris lumbricoides* 1 (0) | 301 (82) |
| Culture and/or PCR for pathogenic enteric bacteria^5^ | 314 | Diarrhoeagenic *E. coli* 10 (3)  *Yersinia* spp. 7 (2)  *Salmonella* spp. 2 (1)  *Campylobacter* spp. 2 (1) | 160 (43) |
| Antigen test for *Giardia / Cryptosporidium*^6^ | 151 | *Cryptosporidium* spp. 3 (2)  *G. lamblia* 2 (1) | 87 (24) |
| Cotton swab sample for enterobiasis | 110 | *Enterobius vermicularis* 1 (1) | 69 (19)^7^ |
| Culture, toxin test or PCR for *Clostridioides difficile* | 61 | *C. difficile* 2 (3) | 29 (8) |
| Antigen test for *Entamoeba histolytica*^8^ | 39 | 0 (0) | 20 (5) |
| Specific sample for *Cryptosporidium spp.* | 23 | 0 (0) | 14 (4) |
| Viral samples^9^ | 4 | 0 (0) | 1 (0) |

Coinfections were found for three patients: *Campylobacter/Giardia*, *EHEC/EPEC,* and *EHEC/Giardia*.

^1^ Enteric pathogens other than *Dientamoeba fragilis.*

^2^ All samples negative for pathogens other than *Dientamoeba* *fragilis* or *Enterobius vermicularis.*

^3^ Ecofix fixation and modified trichrome staining.

^4^ One patient diagnosed with faecal PCR not included in the table.

^5^ PCR assay described by Antikainen et al, 2013; does not cover *C. difficile*.

^6^ Prospect Giardia/Cryptosporidium Microplate Assay, Oxoid Ltd., Basingstoke, United Kingdom.

^7^ One patient tested positive for enterobiasis.

^8^ Entamoeba Celisa Path Test Kit, CeLLabs Pty Ltd., Sydney, Australia.

^9^ Including screening panel for viral antigens, norovirus PCR, rotavirus antigen test, or adenovirus antigen test.

**Supplementary Table 3.** Number of apathogenic intestinal parasite findings for 369 *Dientamoeba fragilis* infection patients.

| **Organism** | **Number of findings, n (%)** |
| --- | --- |
| *Blastocystis hominis* | 122 (33) |
| Multiple apathogens^1^ | 59 (16) |
| *Endolimax nana* | 21 (6) |
| *Entamoeba coli* | 5 (1) |
| *Entamoeba hartmannii* | 1 (1) |

^1^ In addition to other parasites included; *Iodamoeba butschlii* and *Chilomastix mesnili*.

**Supplementary Table 4.** *Dientamoeba fragilis* infection: treatment groups, demographics, control sample timing, and dosage data.

|  | **Doxycycline** | **Metronidazole** | **Paromomycin** | **Secnidazole** | **P-value** |
| --- | --- | --- | --- | --- | --- |
| All episodes, n | 32 | 84 | 297 | 79 |  |
| Female gender, n (%) | 19 (60) | 61 (73) | 193 (65) | 46 (58) | 0.244^1^ |
| Median age, years (IQR) | 45 (35–64) | 40 (16–52) | 42 (30–56) | 9 (5–13) | < 0.001^2^ |
| Episodes for < 18 years, n (%) | 1 (3) | 22 (26) | 39 (13) | 69 (87) | < 0.001^1^ |
| Non-Finnish, n (%) | 7 (22) | 9 (11) | 31 (10) | 3 (4) | 0.039^1^ |
| Foreign travel, n (%) | 20 (63) | 56 (67) | 203 (68) | 37 (47) | 0.005^1^ |
| Apathogenic parasites | 19 (59) | 38 (45) | 180 (61) | 28 (35) | 0.001^1^ |
| Pre-existing conditions, n (%) |  |  |  |  |  |
| Any | 19 (59) | 38 (45) | 133 (45) | 22 (28) | 0.010^1^ |
| GI-related | 6 (19) | 12 (14) | 38 (13) | 7 (9) | 0.517^1^ |
| Control samples |  |  |  |  |  |
| 1^st^ sample, median days from last dose (IQR) | 38 (27–54) | 33 (25–51) | 27 (21–38) | 34 (25–49) | < 0.001^2^ |
| 2^nd^ sample, median days from last dose (IQR) | 49 (30–77) | 44 (29–71) | 51 (29–80) | 45 (32–65) | 0.471^2^ |
| Dosage |  |  |  |  |  |
| Duration, median days (range) | 10 (7–10) | 10.0 (3–14) | 7.0 (2–21) | 1.0 (1–3) |  |
| Dose, median mg (range) | 100 (100–100) | 400 (150–800) | 500 (100–750) | 1000 (300–2000) |  |
| Doses per day, median (range) | 2.0 (1–2) | 3.0 (1–4) | 3.0 (2–5) | 1.0 (1–1) |  |
| Previous courses (%) |  |  |  |  |  |
| No previous courses | 19 (60) | 61 (73) | 102 (34) | 46 (58) | < 0.001^1^ |
| One | 11 (34) | 15 (18) | 109 (37) | 25 (32) |  |
| Two | 2 (6) | 5 (6) | 47 (16) | 4 (5) |  |
| Three | - | 2 (2) | 25 (8) | 3 (4) |  |
| Four | - | - | 10 (3) | 1 (1) |  |
| Five | - | 1 (1) | 2 (1) | - |  |
| Six | - | - | 2 (1) | - |  |
| Days from previous course, median (IQR) | 70 (50–115) | 86 (57–146) | 94 (60–192) | 121 (77–257) | 0.33^3^ |

^1^ *χ^2^* test.

^2^ Kruskal-Wallis test.

^3^ Independent samples median test.

**Supplementary Table 5.** Clearance rates of doxycycline, metronidazole, paromomycin, and secnidazole in *Dientamoeba fragilis* infection treatment with previous courses.

| **Previous courses, n** | **n (%)** | **Faecal clearance, n (%)** | **p-value (*χ^2^)*** |
| --- | --- | --- | --- |
| Doxycycline | 32 | 7 (22) |  |
| None | 19 (60) | 4 (21) | p = 0.6 |
| One | 11 (34) | 2 (18) |  |
| Two | 2 (6) | 1 (50) |  |
| Metronidazole | 84 | 35 (42) |  |
| None | 61 (73) | 26 (43) | p = 0.75 |
| One | 15 (18) | 7 (47) |  |
| Two | 5 (6) | 1 (20) |  |
| Three | 2 (2) | 1 (50) |  |
| Five | 1 (1) | 0 (0) |  |
| Paromomycin | 297 | 247 (83) |  |
| None | 102 (34) | 78 (77) | p = 0.25 |
| One | 109 (37) | 95 (87) |  |
| Two | 47 (16) | 42 (89) |  |
| Three | 25 (8) | 21 (84) |  |
| Four | 10 (3) | 8 (80) |  |
| Five | 2 (1) | 2 (100) |  |
| Six | 2 (1) | 1 (50) |  |
| Secnidazole | 79 | 29 (37) |  |
| None | 46 (58) | 20 (44) | p = 0.39 |
| One | 25 (32) | 8 (32) |  |
| Two | 4 (5) | 0 (0) |  |
| Three | 3 (4) | 1 (33) |  |
| Four | 1 (1) | 0 (0) |  |

**Supplementary Table 6.** Pairwise comparisons of doxycycline, metronidazole, paromomycin, and secnidazole, with significance assessed by *χ^2^* test. Significance level was Bonferroni adjusted to 0.008 (in total 6 comparisons per group).

|  | **Treatment episodes** | | | | | |
| --- | --- | --- | --- | --- | --- | --- |
|  | **All** | | **Adults** | | **< 18 years** | |
| **Regimen pair** | Clearance rate, % | p-value | Clearance rate, % | p-value | Clearance rate, % | p-value |
| Doxycycline vs. metronidazole | 22 vs. 42 | 0.047 | 23 vs. 32 | 0.332 | 0 vs. 68 | 0.348^1^ |
| Doxycycline vs. paromomycin | 22 vs. 83 | < 0.001 | 23 vs. 86 | < 0.001 | 0 vs. 62 | 0.400^1^ |
| Doxycycline vs. secnidazole | 22 vs. 37 | 0.130 | 23 vs. 20 | 1.000^1^ | 0 vs. 39 | 1.000 |
| Metronidazole vs. paromomycin | 42 vs. 83 | < 0.001 | 32 vs. 86 | < 0.001 | 68 vs. 62 | 0.604^1^ |
| Metronidazole vs. secnidazole | 42 vs. 37 | 0.517 | 32 vs. 20 | 0.713^1^ | 68 vs. 39 | 0.017 |
| Paromomycin vs. secnidazole | 83 vs. 37 | < 0.001 | 86 vs. 20 | < 0.001 | 62 vs. 39 | 0.025^1^ |

^1^ P-values by *2-sided* *Fisher’s Exact* test.
